# Socioeconomic inequalities related to perceived difficulty in accessing health services among older adults: A cross-sectional analysis of SABE Study Data

**DOI:** 10.1371/journal.pone.0322333

**Published:** 2025-05-28

**Authors:** Elaine Cristina Tôrres Oliveira, Marília Cristina Prado Louvison, Yeda Aparecida de Oliveira Duarte, Fabíola Bof de Andrade

**Affiliations:** 1 Faculty of Public Health, University of São Paulo (USP), São Paulo, São Paulo, Brazil; 2 State University of Health Sciences of Alagoas, Maceió, Alagoas, Brazil; 3 Faculty of Public Health, Coordinator of the Health, Well-Being and Aging Study (SABE), School of Nursing, University of São Paulo (USP), São Paulo, São Paulo, Brazil; 4 René Rachou Institute, Oswaldo Cruz Foundation (FIOCRUZ), Belo Horizonte, Minas Gerais, Brazil; Universidade Federal de Santa Maria, BRAZIL

## Abstract

Analysis of trends and the magnitude of inequalities in access to health services contributes to identifying privileged groups and facilitates discussions on equity policies. Brazil has an important context for studying healthcare access inequalities due to its rapid population aging and the existence of a universal healthcare system guided by equity principles. Therefore, this study aimed to assess socioeconomic inequalities in the prevalence of difficulties accessing healthcare services among older adults living in the city of São Paulo, Brazil. This cross-sectional study used data from the Health, Well-being, and Aging Study from three years: 2006 (n = 1,386), 2010 (n = 1,319), and 2015 (n = 1,218). The dependent variable used in this study was difficulty accessing healthcare services, an outcome that aimed to assess, based on the perception of access barriers, any difficulty in using or accessing healthcare services when needed. Independent variables were socioeconomic position measured by years of education (which reflects the number of years of education completed) and monthly income (measured in minimum wages). Absolute (SII) and relative (RII) inequality indices were employed to assess the magnitude of socioeconomic inequalities. The findings indicate that individuals with higher socioeconomic status (measured by education and income) experienced lower frequencies of difficulty in accessing healthcare services. Absolute inequalities based on education and income were significant in 2006 [SII:-0.328 (-0.437; -0.220) and SII:-0.191 (-0.295; -0.087), respectively] and 2015 [SII = -0.198 (-0.314; -0.082) and SII = -0.313 (-0.425; -0.201), respectively]. Relative inequalities were significant across all study years, with difficulties in access being 68.0%, 17.0%, and 42.0% lower among individuals with higher education, and 49.0%, 22.0%, and 58.0% lower among those with higher income in 2006, 2010, and 2015, respectively. This study showed that difficulties to access were more concentrated among individuals with lower socioeconomic status, emphasizing the importance of true universalization of the healthcare system to ensure equitable access.

## Introduction

Reducing inequalities represents a global challenge that needs to be strongly addressed [1,2]. Socioeconomic disparities within and between countries into healthcare, resulting in inequities in the access to services [3]. Discussions on tackling inequalities have been central to global agendas, aiming not only identify the factors that create disparate opportunities among individuals but also to develop strategies for progressively achieving greater equity [1].

In the realm of health, access to healthcare services can mitigate inequalities, provided that healthcare systems facilitate access based on individuals’ needs. Access to services and meeting these needs positively impact health and quality of life, underscoring the pivotal role of organizing healthcare systems and policies that prioritize the elimination of inequalities [2].

Many countries have implemented healthcare policies aimed at reducing disparities in access to healthcare services [4–6]. Despite these efforts, inequalities persist [4,7–10], and some studies indicate that socially determined disparities may even be increasing [2,6,10].

Access to healthcare services is a complex concept influenced by factors on both the demand and supply sides [11], and the inequalities observed in access reflect the complex interplay between healthcare systems, individual characteristics, and perceived needs [12]. Factors such as availability, funding, and organization of services play crucial roles in facilitating or impeding access [13]. Additionally, individual differences can act as barriers, creating inequitable opportunities for accessing services [14].

Among the individual factors analyzed in terms of healthcare access, socio-economic conditions significantly contribute to the development of inequalities [15–18]. Studies examining income and education as socio-economic indicators have shown disparities favoring the more privileged groups in access to healthcare services [2,15,18]. For instance, a European Union report analyzing health inequalities across 33 countries from 2014 to 2017 identified a positive income-related inequality gradient. It found that in 75% of the countries studied, individuals with higher incomes were more likely to access or use medical services compared to those with lower incomes, even when facing similar healthcare needs [2]. Regarding education, a systematic review underscored an association between education levels and access to medical consultations, observed across low, middle-income, and high-income countries [18].

The analysis of the context of inequalities in access to healthcare services among older adults has also revealed disparities depending on the individual’s socioeconomic status. A study conducted in Chile with individuals aged 60 and older found the presence of inequalities (pro-rich) in access to medical consultations [19]. This finding was similarly identified in a cohort study conducted in China, which observed an association between deprivation in socioeconomic status and inequality in access to medical services among older adults [5].

Disparities in access to healthcare services persist even in countries with universal healthcare systems [7–9,20,21]. In Brazil, despite the implementation of the Unified Health System (SUS), a mixed public-private system, coupled with territorial and social inequalities continues to affect healthcare access. Analyses conducted by the National Health Survey (PNS) in 2019 revealed that the number of individuals who had access to a medical appointment in Brazil increased as income levels rose [20]. Similarly, data from the National Household Sample Survey (PNAD) from 1998 to 2013 and subsequent PNS data from 2013 highlighted a pattern of greater utilization of medical and dental services among individuals with higher levels of education [21].

Studies on inequalities in access to healthcare services are essential because they signal to healthcare systems the elements that create barriers and difficulties in obtaining care [18]. Furthermore, analyzing the trends and magnitude of these inequalities contributes to identifying privileged groups over time. This identification enables discussions and facilitates the development and evaluation of policies aimed at promoting equity [22].

Most analyses of the magnitude of inequalities, utilizing complex measures, have focused on the adult population, highlighting a need to understand the impact of socioeconomic inequalities on difficulties in accessing healthcare services by older adults. Older adults have higher healthcare service utilization, emphasizing the importance of comprehensive planning to meet their needs and mitigate potential inequalities [23].

Brazil is an important study context for examining inequalities in access to healthcare services among the older adults, given its rapidly aging population [24] and the presence of a universal healthcare system grounded in principles of equity [25]. Therefore, this study aimed to assess socioeconomic inequalities in the prevalence of difficulties accessing healthcare services among older adults living in the city of São Paulo, Brazil.

## Materials and methods

This is a cross-sectional study with data from the Health, Well-being, and Aging Study (SABE Study), collected in the years 2006, 2010, and 2015. The SABE Study aims to assess the health status of older adults and facilitate dialogue between public health research and aging studies. The target population comprises individuals aged 60 and over of both genders, non-institutionalized, living in urban areas of the city of São Paulo, which hosts the largest absolute number of older adults in Brazil [26]. The concept of older adults used in this study aligns with Brazilian legislation defining individuals aged 60 years or older as older adults [27].

In Brazil, the SABE Study (originally initiated by the Pan American Health Organization) evolved into a longitudinal study comprising multiple cohorts. Participants were first interviewed in the year 2000 and reevaluated in 2006, 2010, and 2015. In each wave, a new sample of individuals aged 60–64 years old was included to keep the representativeness of this age group. New weights were calculated at every wave to ensure representativeness of the São Paulo population in terms of age and study year [28]. The sampling process utilized census tracts as primary units of analysis and randomly selected households as secondary units. Further details regarding the sampling methodology can be found in other publications [26,29,30].

The original sample sizes for the 2006, 2010, and 2015 cohorts were 1,413, 1,345, and 1,224 individuals, respectively. For the present study, the analysis included participants with complete information on the variables of interest, resulting in sample sizes of 1,386, 1,319, and 1,218 individuals for the years 2006, 2010, and 2015, respectively. Participants were recruited during the following periods: March 31, 2006, to August 19, 2007; October 12, 2010, to December 16, 2011; and March 18, 2016, to July 29, 2018.

The dependent variable was the perceived difficulty in accessing healthcare services was designated as the dependent variable and assessed using the question: “*Do you have any difficulty in using or accessing healthcare services when needed?*” Response options included yes, no, don’t know, and/or no response, with yes or no responses selected for analysis. The perceived difficulty of access by older adults was used to value their subjective perception of barriers encountered in fulfilling their healthcare needs [30].

Two socioeconomic position variables were considered as independent variables: years of education and monthly income. The years of education variable reflects the number of years of education completed by the participants and was categorized into five groups (less than 1 year, 1–3 years, 4–7 years, 8–11 years, and 12 or more years). Monthly income was measured in minimum wages and categorized into six groups (no income, up to 1 minimum wage, 1.01 to 2.00 minimum wages, 2.01 to 3.00 minimum wages, 3.01 to 5.00 minimum wages, and 5.01 or more minimum wages). The following variables were used for descriptive porpoises: gender (male, female) and age group (60–69 years, 70–79 years, 80 years and older).

Descriptive analyses were conducted by presenting frequencies and calculating the corresponding 95% confidence intervals for all study years. Following the sample description, bivariate analysis was conducted using the Pearson chi-square test with Rao-Scott correction (considering for sample weights) to examine the association between the outcome (difficulty in accessing healthcare services) and the independent variables (gender, age group, and measures of socioeconomic position). The distribution of difficulty in accessing healthcare services based on socioeconomic position measures was depicted using the *Equiplot*. The *Equiplot* graph is highly effective for illustrating how inequalities impact health conditions by comparing different groups relative to the same indicator. Its visualization facilitates the identification of each group’s situation concerning the indicator and visually conveys the magnitude of inequalities between groups (http://www.equidade.org/equiplot.php).

The magnitude of socioeconomic inequalities related to difficulty in accessing healthcare services was assessed using complex inequality measures: the *Slope Index of Inequality* (SII) and the *Relative Index of Inequality* (RII). Both indices consider the entire socioeconomic distribution in the analysis rather than comparing extreme groups. They are obtained through regression of the outcome variable (difficulty in accessing healthcare services) on a relative position score derived from socioeconomic position measures (years of education and income). The relative position score was generated by ranking the sample in ascending order based on socioeconomic position, where a score of zero represents the lowest socioeconomic position (e.g., “less than 1 year of education = 0” and “no income = 0”), and a score of one represents the highest socioeconomic position (e.g., “12 or more years of education = 1” and “5.01 or more minimum wages = 1”). Each socioeconomic position group was assigned a value corresponding to the midpoint of its cumulative distribution within the analyzed categories. This relative position variable was then included as an independent variable in the regression model [31–34].

The SII represents the absolute difference in the probabilities of experiencing difficulty in accessing healthcare services between those in the highest and those in the lowest socioeconomic position scores. A negative SII indicates a higher probability of difficulty in accessing healthcare services among individuals with lower socioeconomic positions. A SII value of zero indicates no inequality in access to healthcare services. [30–33].

The RII expresses the ratio between the probabilities of experiencing difficulty in accessing healthcare services among groups with the highest and lowest socioeconomic positions. A RII less than one indicates a higher probability of difficulty in accessing healthcare services among individuals in the lower socioeconomic position group. A RII value of one signifies no inequality in access to healthcare services [30–33]. Because the outcomes of SII and RII are binary, both are estimated using logistic regression to ensure predictions fall within the range of 0 and 1 [34].

Data analysis was conducted using Stata 15 software, employing the *svy* module to account for the complexity of the sampling design.

### Ethical aspects

All the participants provided consent in writing after receiving explanations about the study. If the elderly person was unable to consent, this action was the responsibility of their guardian. Informed consent was obtained directly during the data collection preceding the interviews. The SABE Study and its ethical procedures were approved by the Research Ethics Committee of the Faculty of Public Health at the University of São Paulo, with approval numbers 83 (2006), 2044 (2010), and 3,600,782 (2015).

## Results

Table 1 presents the distribution of the sample by sociodemographic characteristics and difficulty in accessing healthcare services. In 2006, 2010, and 2015, the categories with the highest proportions of participants were female (60.0%, 59.8% and 56.8%, respectively), aged 60–69 (58.4%, 53.6% and 54.4%, respectively) and with 4–7 years of schooling (38.6%, 37.6% and 37.4%, respectively). Regarding monthly income, the distribution varied across study periods, with higher proportions observed between 1.01 and 2.00 minimum wages in 2006 (29.1%) and 2015 (27.1%), and up to one minimum wage in 2010 (48.0%). In terms of difficulty accessing healthcare services, the lowest proportion was reported in 2010 (24.9%), while the highest was in 2015 (37.3%) (Table 1).

**Table 1:** Distribution of the sample by sociodemographic variables and difficulty in accessing health services. SABE study, São Paulo, 2006, 2010 and 2015.

Variables	2006 (n = 1,386)	2010 (n = 1,319)	2015 (n = 1,218)
	% (95% CI^a^)	% (95% CI^a^)	% (95% CI^a^)
**Sex**			
Male	40.0 (37.0, 43.1)	40.1 (37.3, 43.1)	43.2 (40.5, 45.8)
Female	60.0 (56.9, 63.0)	59.8 (56.9, 62.7)	56.8 (54.1, 59.4)
**Age group**			
60 to 69 years	58.4 (51.9, 64.6)	53.6 (45.2, 61.9)	54.4 (46.2, 62.3)
70 to 79 years	30.4 (25.6, 35.6)	31.0 (25.4, 37.3)	30.5 (24.3, 37.4)
80 and over	11.2 (8.1, 15.2)	15.3 (11.8, 19.6)	15.1 (11.5, 19.7)
**Years of study**			
Less than 1 year	15.8 (12.9, 19.2)	12.0 (9.5, 15.0)	13.0 (10.8, 15.6)
1 to 3 years	26.7 (23.7, 30.1)	22.5 (19.3, 26.1)	17.9 (15.4, 20.7)
4 to 7 years	38.6 (35.1, 42.2)	37.6 (34.3, 41.1)	37.4 (34.2, 40.7)
8 to 11 years	12.7 (10.5, 15.4)	18.6 (15.0, 22.7)	20.2 (17.6, 23.0)
12 and more	6.2 (4.2, 9.0)	9.3 (6.7, 12.7)	11.4 (8.7, 15.0)
**Income** ^b^			
No income	3.7 (2.7, 5.2)	10.4 (8.5, 12.7)	8.2 (6.6, 10.3)
Up to 1.00	23.1 (20.0, 26.5)	48.0 (43.5, 52.4)	22.7 (19.4, 26.4)
From 1.01 to 2.00	29.1 (25.5, 33.1)	18.1 (15.1, 21.6)	27.1 (23.9, 30.6)
From 2.01 to 3.00	13.6 (11.4, 16.2)	9.2 (7.4, 11.3)	16.8 (14.2, 19.7)
From 3.01 to 5.00	16.0 (13.3, 19.2)	7.8 (6.0, 10.1)	14.1 (12.0, 16.6)
From 5.01 and more	14.4 (11.7, 17.6)	6.5 (4.7, 9.0)	11.0 (8.7, 13.7)
**Difficulty in accessing health services**			
No	70.4 (66.5, 74.0)	75.1 (71.3, 78.6)	62.7 (58.4, 66.7)
Yes	29.6 (26.0, 33.5)	24.9 (21.4, 28.7)	37.3 (33.2, 41.6)

^a^95% CI: 95% Confidence Interval.

^b^Value of the minimum wage at the time of data collection taken as a reference was: 2006: 350.00 reais (about 161.29 US dollars), 2010: 622.00 reais (about 296 US dollars), 2015: 880.00 reais (about 225 US dollars).

Table 2 presents the differences in difficulty accessing health services according to the study period. There was a significant association between difficulty in accessing health services and gender in both 2006 (p = 0.045) and 2015 (p < 0.001). In 2006, the proportion of access difficulties was 31.5% among women and 26.2% among men. By 2015, these proportions had increased to 42.2% among women and 30.9% among men.

**Table 2:** Bivariate analysis for the difficulty in accessing health services among older adults by gender and age group. SABE study, São Paulo, 2006, 2010 and 2015.

Variables	Difficulty in access		
	2006	2010	2015
	% (95% CI^a^)	% (95% CI^a^)	% (95% CI^a^)
**Sex**			
Male	26.6 (21.9, 32.0)	22.9 (18.5, 28.0)	30.9 (25.4, 37.0)
Female	31.5 (27.8, 35.5)	26.2 (22.5, 30.4)	42.2 (37.9, 46.6)
p-value	0.045	0.150	<0.001
**Age group**			
60 to 69 years	29.6 (24.5, 35.4)	26.4 (20.7, 33.1)	41.1 (35.5, 46.8)
70 to 79 years	27.0 (22.1, 32.5)	22.7 (17.5, 29.0)	35.2 (27.8, 43.4)
80 years and over	36.4 (30.9, 42.2)	23.9 (19.3, 29.2)	28.1 (21.0, 36.5)
p-value	0.168	0.541	0.059

^a^95% CI: 95% Confidence Interval.

**Fig 1:**
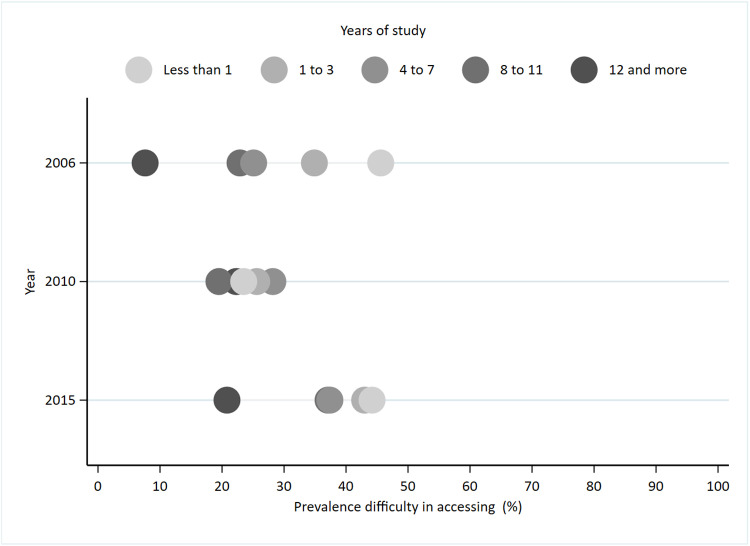
Prevalence of difficulty in accessing health services according by years of schooling. SABE study, São Paulo, 2006, 2010 and 2015.

**Fig 2:**
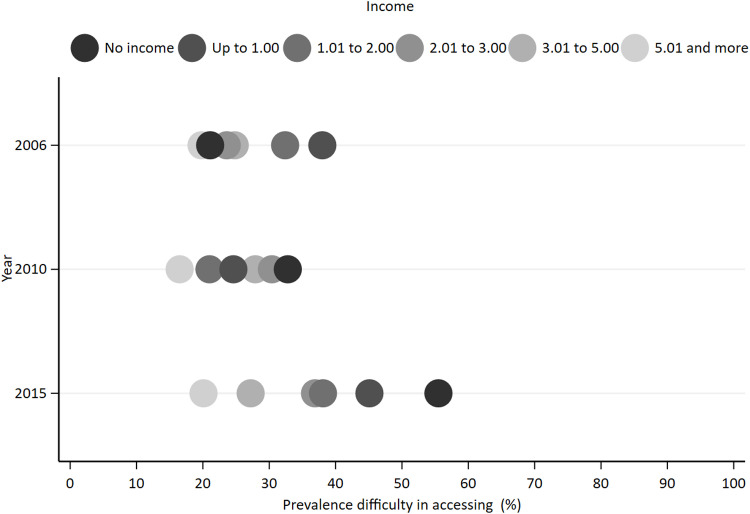
Prevalence of difficulty in accessing health services by income. SABE study, São Paulo, 2006, 2010 and 2015.

Figs 1 and 2 graphically depict the prevalence of difficulty in accessing health services according by socioeconomic position measures in 2006, 2010, and 2015. The *Equiplot* visually identifies the prevalence within each socioeconomic group and illustrates the absolute inequality between these groups. Overall, individuals with higher socioeconomic positions, as measured by both years of education and income, had lower prevalence of difficulty in accessing health services. However, distinct patterns were observed across the study years.

There was an association between years of schooling and difficulty in accessing health services in 2006 and 2015. In 2006, the proportion of individuals experiencing difficulty in accessing health services was 45.6% (95% CI: 36.4, 55.1) among those with less than one year of schooling, and to 7.6% (95% CI: 3.1, 17.6) among those with 12 years or more of schooling. By 2015, these proportions were 44.2% (95% CI: 35.1, 53.7) for individuals with less than one year of schooling and 20.7% (95% CI: 13.7, 30.2) for those with 12 years or more (Fig 1).

There was a significant association between income levels and difficulty in accessing health services both 2006 and 2015. In 2006, 38.0% (95% CI: 31.1, 45.4) of the participants earning up to one minimum wage reported difficulty accessing services, and 19.3% (95% CI: 12.1, 29.3) among those earning more than five minimum wages. By 2015, the proportion increased to 55.5% (95% CI: 43.8, 66.6) among those with no income, while it was 20.1% (95% CI: 12.9, 30.0) among those with a monthly income of five or more minimum wages (Fig 2).

In 2010, lower levels of difficulty in accessing health services were observed across categories of both years of schooling and income (Figs 1 and 2). The proportion experiencing difficulty in accessing health services was 23.5% (95% CI: 16.9, 31.6) for those with less than one year of schooling and 22.3% (95% CI: 13.6, 34.2) for those with 12 years or more of schooling. Regarding income, 31.5% (95% CI: 22.8, 41.8) of the individuals earning up to one minimum wage reported difficulty accessing services, while 16.5% (95% CI: 9.6, 26.8) of those earning more than five minimum wages faced similar challenges. However, there was no statistically significant association between difficulty of access and measures of socioeconomic position in 2010.

Table 3 shows the absolute (SII) and relative (RII) inequalities related to difficulty in accessing health services. In terms of absolute measures, in 2006, the probability of difficulty in accessing health services was 0.33 percentage points (p.p) lower among individuals with higher levels of education [SII: -0.328 (-0.437, -0.220)] and 0.19 p.p lower among those with higher income [SII: -0.191 (-0.295, -0.087)]. Similarly, in 2015, the findings indicate a lower probability of difficulty in accessing health services among individuals with higher levels of schooling and income [schooling SII = -0.198 (-0.314, -0.082), income SII = -0.313 (-0.425, -0.201)].

**Table 3:** Absolute (SII) and relative (RII) inequalities related to difficulty in accessing health services by socioeconomic status. SABE study, São Paulo, 2006, 2010 and 2015.

Years of analysis	Years of study	Income	Years of study	Income
	SII^a^ (95% CI^c^)	SII^a^ (95% CI^c^)	RII^b^ (95% CI^c^)	RII^b^ (95% CI^c^)
2006	−0.328^A^ (−0.437, −0.220)^d^	−0.191^AB^ (−0.295, −0.087)^d^	0.32^A^ (0.190, 0.450)^d^	0.51^AB^ (0.320, 0.710)^d^
2010	−0.047^B^ (−0.145, 0.051)	−0.063^B^ (−0.166, 0.040)	0.83^B^ (0.500, 1.160)^d^	0.78^B^ (0.460, 1.100)^d^
2015	−0.198^AB^ (−0.314, −0.082)^e^	−0.313^A^ (−0.425, −0.201)^d^	0.58^B^ (0.390, 0.780)^d^	0.42^A^ (0.280, 0.560)^d^

^a^SII: *Slope index inequality*

^b^RII: Relative index inequality.

^c^95% CI: 95% Confidence Interval.

^d^Statistically significant association (p < 0.001)

^e^Statistically significant association (p = 0.001)

^A^,

^B^,

^AB^: equal letters represent that there was no difference between the years studied.

Relative inequalities were significant in all years of study. In 2006, difficulty in accessing health services was 68.0% lower among individuals with higher levels of schooling, 17.0% in 2010 and 42.0% in 2015. Similarly, in terms of income, the difficulty of access was lower among individuals with higher socioeconomic status (49.0% in 2006, 22.0% in 2010, and 58.0% in 2015) (Table 3).

Regarding relative inequalities (RII), there were significant changes observed. For schooling, there was a reduction in difficulty of access between 2006 and 2010 and also between 2006 and 2015, indicating a decrease in relative disparities. In contrast, for income, there was an increase in difficulty of access between 2010 and 2015, highlighting a rise in relative disparities in access to health services during this period (Table 3).

## Discussion

The analysis of inequalities in access to health services plays a crucial role in shaping health systems and policies aimed at achieving equity. This study focused on Brazil, specifically examining disparities among older adults. The findings suggested significant, absolute and relative socioeconomic inequalities in self-reported difficulty in accessing healthcare services. Individuals with higher socioeconomic status, whether measured by education or income, had lower frequency of difficulty in accessing healthcare services. Moreover, the study revealed distinct patterns of change in absolute (SII) and relative (RII) inequalities across the socioeconomic measures analyzed. There was a reduction in both SII and RII concerning years of education, indicating a narrowing of disparities in healthcare access. Conversely, measures related to income showed an increase in both SII and RII, suggesting a widening gap in healthcare access between income groups.

The findings of this study are consistent with previous research that highlighted disparities in access to healthcare services based on socioeconomic factors such as education [5,35–37] and income [2,20,38,39]. A study using data from the 2013 National Health Survey (PNS) in Brazil found that individuals with higher education had significantly better access to healthcare compared to those with no education [36]. Similarly, findings related to income levels align with analyses from the 2019 PNS, which demonstrated a progressive increase in access to medical consultations as household income levels rose [20]. Moreover, another study using the same dataset revealed higher rates of unmet health needs among lower-income individuals than to their higher-income counterparts [38].

In this study, significant socioeconomic inequalities were observed favoring individuals who are socioeconomically more vulnerable. However, the factors contributing to these inequalities may vary depending on the specific socioeconomic measure employed. It is important to point out that schooling may not be an isolated characteristic of socioeconomic status [37]. Higher levels of education often correlate with better job opportunities, higher income levels, and greater access to information, which in turn influence health-related habits and behaviors [5,37].

Furthermore, the influence of socioeconomic status, particularly income, on the ability to access healthcare services when needed is a significant concern, especially in São Paulo, the city with the highest absolute number of older adults in Brazil. Despite the presence of a public and universal healthcare system, which theoretically ensures equitable care without discrimination [40], disparities linked to income persistently affect healthcare access among older adults.

This study builds on the existing literature by examining changes in healthcare access inequalities across three distinct time points. The findings indicate a decrease in both absolute and relative disparities concerning educational attainment, while conversely highlighting an increase in disparities associated with income levels. These results necessitate a nuanced interpretation that considers the role of Brazil’s Unified Health System (Sistema Único de Saúde, SUS), as well as the distinct impacts of each socioeconomic measure on healthcare treatment needs and access. Moreover, it is crucial to analyze the transition dynamics among different socioeconomic groups over time.

In Brazil, efforts to ensure equitable access to healthcare have been anchored in the establishment and evolution of the Unified Health System (Sistema Único de Saúde, SUS) since its regulation in 1990. The SUS embodies the constitutional right to health and has progressively implemented a range of individual and collective health interventions aimed at addressing the determinants and conditions influencing health outcomes, including educational attainment [40]. The SUS operates on principles that prioritize universal access and equity in healthcare delivery. These principles are fundamental in addressing inequalities linked to educational disparities.

The inverse equity hypothesis offers a nuanced perspective on the dynamics of healthcare access and socioeconomic inequalities over time. According to this hypothesis, public health interventions initially prioritize individuals of higher socioeconomic status, potentially exacerbating inequalities in the short term. However, as these interventions mature and become more inclusive, they eventually extend their benefits to individuals of lower socioeconomic status, thereby reducing overall disparities [41]. In the context of this study, the graphical analysis of the prevalence of difficulty in accessing healthcare services, stratified by years of education, suggests that the reduction in the distance between individuals with the lowest and highest schooling scores may reflect a stabilization of difficulty in access among those with less schooling, coupled with an increase in difficulty of access among those with higher levels of education.

The study also reveals a concerning trend regarding income-related disparities in accessing healthcare services over the study periods. Contrary to the observed reduction in inequalities related to education, both absolute and relative measures indicate an increase in difficulties accessing services among individuals with lower incomes. This trend is particularly worrisome in light of Brazil’s aging population and the rising burden of chronic diseases that contribute to multimorbidity among this age group [42]. A comparative analysis of the *Equiplot* between study years reveals notable changes in the distribution of difficulty in accessing health services across socioeconomic groups. Specifically, the increase in distance observed between extreme groups might be explained by the stability of the difficulty of access among the richest and the increase in the difficulty of access among the poorest.

The coexistence of multiple health subsystems in Brazil, despite the presence of a universal healthcare system maintained by the state, significantly contributes to disparities and inequalities in access to health services. While Brazil’s Unified Health System (SUS) aims to provide equitable healthcare access to all citizens, the expansion of private healthcare companies and health insurance plans has introduced complexities that undermine the universal healthcare agenda [43].

The increased difficulty of accessing healthcare services observed in this study from 2006 to 2015 may be attributed to a combination of factors related to both the supply of healthcare services and the demand presented by the population. It is noteworthy that during this period, there was a notable expansion in the number of healthcare establishments registered and providing services across São Paulo, encompassing public, private, philanthropic, and union-operated facilities. Specifically, data reveals a significant increase of approximately 100% in the number of healthcare establishments in São Paulo municipality [44]. This finding reinforces the understanding that the availability of health services is an important aspect of access but is not in itself the only element responsible for guaranteeing it [45,46].

Aspects related to demand, such as those resulting from predisposing factors, enabling factors and health needs, are closely linked to obtaining goods and services [14] and may have contributed to the difficulty in access experienced by the population in this study. In this sense, investigating aspects related to the individual makes it possible to understand which factors, beyond supply, constitute barriers to accessing health services in the study scenario.

It is important to highlight that the year 2010 presented the lowest distributions of difficulty in accessing health services, both for education and income. This scenario may be related to the implementation of planning strategies observed in Brazil from 2003 onwards. There was an intensification of national health planning strategies and instruments, aiming to give a new direction to health policy in the country, strengthening the technical-political and social, with the involvement of various actors [47]. Government actions developed between 2003 and 2010, marked by a democratic, managerial and developmental orientation, may have had an impact on access to healthcare services and reduced inequalities.

Although this study sought to understand socio-economic inequalities, the study has some limitations. It should be noted that, as the survey was carried out between 2006 and 2015, its results may not represent the current situation in the municipality of São Paulo, mainly due to the Covid-19 pandemic. In addition, the difficulty in access was assessed through the self-perception, which can be influenced by culturally incorporated concepts and models of health care. Another important point is that the possible simultaneous use of health subsystems (public and private) among individuals was not considered, which may generate a greater likelihood of accessing services among those who are more privileged.

## Conclusion

This study identified the existence of socioeconomic inequalities concerning the difficulty in accessing healthcare services among older adults, producing disparities in favor of the more privileged in Brazil. It was found that during the study period, inequalities related to schooling decreased between 2006 and 2010 and 2006 and 2015, while inequalities related to income increased between 2010 and 2015.

The analysis of inequalities carried out by this study highlights the importance of using absolute and relative measures and the prevalence of the outcome to better understand the scenario studied [32]. The findings observed strengthen the understanding of the social production of health inequalities, highlight the importance of maintaining efforts to strengthen universal health systems and contribute to updating social policies that seek to ensure equity in access.

Given the results, there is a need to monitor inequalities to verify their and their magnitude and trends, given the implementation of social policies.

## Supporting information

S1 File Minimal data set. Set of data analysed for each individual include in this study.
(XLSX)
